# Breakthrough in the Development of Endodontic Irrigants

**DOI:** 10.7759/cureus.66981

**Published:** 2024-08-16

**Authors:** Kapil Naladkar, Manoj Chandak, Swayangprabha Sarangi, Paridhi Agrawal, Namrata Jidewar, Tejas Suryawanshi, Palak Hirani

**Affiliations:** 1 Department of Conservative Dentistry and Endodontics, Sharad Pawar Dental College and Hospital, Datta Meghe Institute of Higher Education and Research, Wardha, IND

**Keywords:** removal of smear layer, irrigation in endodontics, ultrasonic device, root canal therapy, irrigants

## Abstract

The three main components of endodontic success are three-dimensional obturation, pulp space sterilization, and biomechanical preparation. Instrumentation techniques are insufficient to accomplish complete disinfection of the pulp space. It is essential to use additional tools, such as endodontic irrigants. This review article aims to provide a general overview of the different root canal irrigants and their clinical uses. Endodontic treatment focuses more on removing infectious material from the root canal system to facilitate the healing of an existing periapical lesion or to prevent the periradicular tissues from being infected. It is important to note that instrumentation alone can reach every part of the root canal wall. Therefore, the irrigation procedure is a crucial aspect of endodontic therapy.

Irrigation requires the application of various irrigants, like sodium and chlorhexidine, to get rid of debris, bacteria, and tissue leftovers. The accomplishment of root canal treatments counts heavily on the thoroughness of irrigation, contributing to optimal canal shaping and disinfection. Modern root canal irrigation systems incorporate syringes, needles, and advanced delivery mechanisms, including sonic and ultrasonic devices. These inventions aim to enhance the mechanical action of irrigants, reaching intricate canal anatomy more efficiently. Understanding the dynamics of root canal irrigation and staying abreast of technological advancements are essential for clinicians to achieve improved treatment outcomes in endodontic procedures.

## Introduction and background

To perform a successful endodontic root treatment, it is necessary to remove bacteria and bacterial toxins thoroughly and any remaining viable and nonviable pulpal tissue from the root canal [1]. While carrying out biomechanical therapy, the irrigation fluids act as lubricants and cleansers, effectively eliminating bacteria and related products associated with tissue degradation [2]. Irrigation is crucial in root canal treatment, particularly for removing pathogens [3]. When ultrasonic energy is used, irrigation helps to break down tissue, improve the cutting efficiency of the file, and reduce the temperature of the file and tooth. Planktonic biofilm bacterial extrusion into the apical region may be inhibited by irrigation [4].

The primary objective of an endodontist is to either prevent an infection from spreading to the periradicular tissue or remove infectious debris and microbes through a root canal. Irrigation depends on the irrigant's mechanism of action and the capacity to interact with the element, material, and structure. Because this dissolves organic matter, removes microbes, serves as a lubricant, and is nontoxic, sodium hypochlorite (NaOCl) is an efficient disinfectant [5]. Chemical irrigators are used in the root canal to dissolve tissue remnants, eliminate microorganisms, and thoroughly clean the area without any negative effects. Several irrigating solutions eliminate microbes when they come in contact with microorganisms because they possess antimicrobial activity. These characteristics should be present in the perfect irrigant solution. It should be capable of dissolving the remnants of nonvital pulp, inactivating endotoxins, and having a broad antimicrobial action [6]. It is verified that the intracanal introduction of the irrigating solution provides analgesic-like action, playing a critical component in pain regulation [7]. It cannot be accomplished alone by biomechanical preparation. Therefore, the use of irrigants is a must [8,9].

## Review

**Figure 1:**
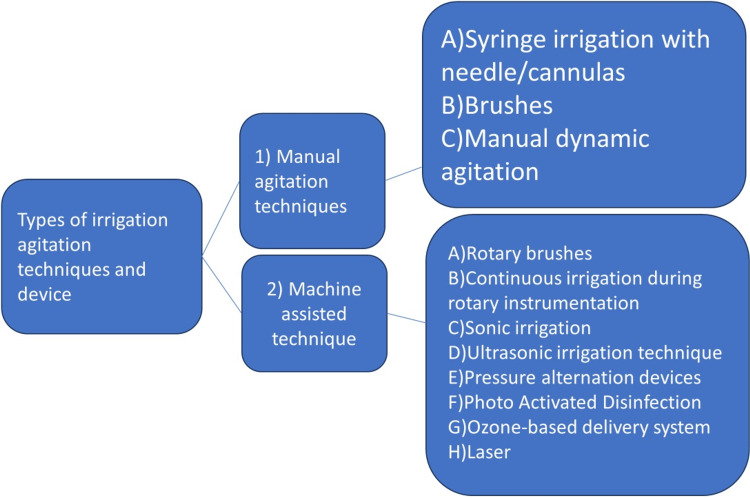
Classification of irrigation agitation techniques and devices

The irrigation agitation techniques and devices can be classified as shown in Figure 1.

Manual agitation technique

Dentists continue to use the manual irrigation technique using needles every day. In this case, a range of gauge needles or cannulas are used to apply the irrigant, which can be applied gently or forcefully [10].

Syringe irrigation with needles/cannulas involves using needles or cannulas with different gauges to dispense an irrigant into a canal. This action can be performed passively or through agitation by pushing and pulling the needle within the canal [11,12]. The size and structure of the irrigation tip greatly affect the flow pattern, speed, depth of reaching, and pressure on the walls and apex of the canal. The gauge of the irrigation tip primarily determines how deeply the irrigant can be introduced into a canal. To carry out a successful endodontic root treatment, it is crucial to effectively eliminate bacteria, bacterial toxins, and remnants of both viable and nonviable pulpal tissue from the root canal [13]. Biomechanical therapy involves using irrigating fluids as lubricants and cleansers to eliminate bacteria and products associated with tissue deterioration [14].

Manual dynamic agitation

In order to work effectively, an irrigant must be closer to the canal walls. Often, the irrigant finds it challenging to access the periapical portion of the canal due to the phenomenon known as the vapor lock effect [15,16]. A snugly fitting gutta-percha cone moved gently upward and downward inside an instrumented canal can enhance the displacement and create an efficient hydrodynamic effect. The elements influencing manual dynamic irrigation include the higher frequency of back-and-forth motion (3.3 Hz) of the gutta-percha point, which leads to greater intracanal pressure changes during pushing movements. This results in more significant fluid movement within the canal than Rinsendo's (Durr Dental Co., Bietigheim-Bissingen, Germany) positive-negative hydrodynamic pressure frequency (1.6 Hz). The latter most likely makes it possible to mix the spent, reacted irrigant more effectively with the fresh, unreacted solution [17].

Machine-assisted agitation technique

The development of manually assisted systems resulted in the evolution of instruments that can slowly rotate into the root canal filled with irrigants using handpieces. Files and other instruments may exhibit a smooth surface with increasing taper or even a surface with lateral extensions of plastic files [18-21]. Ruddle has a rotary handpiece-attached microbrush to help remove dentinal debris from root canals. Microbrush spins at roughly 300 revolutions/minute. These brushes are not meant to irrigate the canal spaces directly. These are planned alternatives for agitating root canal irrigants. Canal Brush (Roeko Canal Brush™, Coltène/Whaledent, Langenau, Germany) is another endodontic microbrush that has just entered the market [22]. This incredibly flexible microbrush can be used manually with a rotating motion and is made entirely of polypropylene. The small, flexible root canal brush with irrigant debris was successfully removed from the canal.

Quantec-E irrigation system

Attached to the Quantec-E Endo System (Sybron Endo, Orange, CA) is the self-containing fluid dispensary unit known as the Quantec-E irrigation system [23]. A greater volume of irrigant would be produced, the irrigant exposure time would increase, and deeper irrigant access inside the root canal would be made possible. Quantec-E irrigation did, according to studies by Walters et al., produce cleaner canal walls and more by removing smear layers and debris from the canal [19,24]. It provides continuous irrigation during instrumentation. During the treatment, it is crucial to maintain a constant flow of irrigant into the root canal rather than delivering it intermittently with a syringe needle. The self-adjusting file (SAF) system is designed for endodontic therapy and minimizes the need for physical intervention during cleaning and shaping procedures. It uses a VATEA irrigation pump and a specialized handpiece head (Rement Dental Technology, Kowloon, Hong Kong) that permits an uninterrupted irrigant flow across the file. There are two diameters available: 1.5 and 2.0. Both are incredibly compressible. The dimensions of the 0.5 and 2.0 mm compressed files are 20k and 35k, respectively [25]. The system features continuous irrigation, effectively flushing the root canal with solutions to remove debris, disinfect, and provide clear visibility. Thanks to its self-adjusting action and continuous irrigation, SAF reduces the time required for root canal procedures compared to traditional methods. Clinical studies have shown that SAF effectively cleans and shapes root canals, leading to high success rates and improved patient outcomes.

Sonic irrigation

Tronstad et al. [17] introduced sonic instruments in 1985. Compared to ultrasonic irrigation, it produces minimal shear stresses and works at a frequency of 1-6 kHz. Numerous sonic irrigation tools are available in the marketplace [26]. Endodontic treatments have a higher success rate by using acoustic streaming to activate the disinfectant, and the irrigation process is completed and enhanced. It breaks the layers of smears and enhances debridement [27]. Compared to the syringe irrigation technique, it shows better irrigation for clearing debris and smears of the canal [28]. Endo activator is a mechanic-based system comprising different polymer tips and a handpiece. The tips are adaptable and tough to break. It dislodges the biofilm within the long, tortuous, narrow canal of teeth. It delivers 10,000 cpm/minute [29-31]. Sonic irrigation in endodontics offers several advantages. It facilitates thorough cleaning and disinfection of the root canal system compared to conventional methods. Sonic energy helps better penetrate the irrigation solution into complex anatomical structures, ensuring the comprehensive removal of debris and bacteria. Additionally, it enhances the removal of contaminants from hard-to-reach areas within the root canal, such as lateral canals and isthmuses, which may be challenging to access using traditional techniques. Moreover, sonic irrigation can reduce instrumentation time, improving efficiency during endodontic procedures.

Ultrasonic irrigation

Ultrasonic oscillates at 25- 30 kHz [32,33]. There are two types of ultrasonics: simultaneous ultrasonic instrumentation and irrigation (UI) and passive ultrasonic irrigation [2]. During ultrasonic continuous irrigation, an irrigant is administered intravenously through tubing that is attached to an irrigation-delivering syringe via a Luer lock (Becton Dickinson, Franklin Lakes, NJ). At the same time, the ultrasonic handpiece activates the needle [34,35]. PUI offers benefits such as improved penetration into complex structures, better debris and bacteria removal, and enhanced disinfection, especially in challenging areas like isthmuses and lateral canals. Success with PUI hinges on factors like case specifics, practitioner skill, and equipment quality. When used properly, PUI helps to improve treatment results by effectively cleansing and disinfecting the root canal system. Weller et al. first used the term "passive ultrasonic irrigation" [31]. This noncutting technique lessens the possibility of irregularly shaped canal development. During PUI, ultrasonic waves transfer energy from a file to the irrigating solution, resulting in two phenomena. The irrigant undergoes cavitation and streaming [36,37]. Like other endodontic techniques, the success of ultrasonic irrigation depends on several variables, such as the particulars of the case, the skill of the clinician, and the caliber of the tools used. When applied correctly, ultrasonic irrigation can significantly contribute to more comprehensive root canal treatment and improved patient results.

Modern root canal disinfection methods combine the conventional CMP method with ultrasonics and lasers. While lasers have been sold commercially in dentistry since 1990, ultrasonic devices were initially launched in endodontics. Dentistry uses lasers for many purposes: caries detection (DIAGNOdent Pen, KaVo Dental, Biberach an der Riss, Germany), pulpal blood flow diagnosis, dentinal hypersensitivity treatment, pulp capping, pulpotomy, smear layer removal, sterilizing root canals, teeth preparation, enamel etching, gingivectomy, bleaching, periodontal pocket disinfection, calculus removal, and laser photosensitization of the root canal [37,38]. Both the dentin's deeper layers and the surfaces of root canals are susceptible to the bactericidal effects of this kind of laser.

Pressure alteration devices

This appears to be associated with traditional syringe needle irrigant delivery. Because of the air trapping, it is challenging to reach the apical third region of the root canal [38]. Concurrent irrigant conveyance and aspiration through pressure alternation devices offer a viable solution to this problem [39]. The EndoVac system (Discus Dental, Culver City, CA) provided apical negative pressure irrigation. The upper two-thirds of the root canal uses a suction method to clear debris and promote irrigation flow. Macrocannula, microcannula, and the tip of master delivery are its three constituent parts. At the same time that it distributes the irrigant, the master delivery tip expels it. The macrocannula is made of plastic and measures 55 mm long. It is on a titanium handle and has a 0.02 taper for coarseness. Negative pressure thus provides a steady supply of new irrigants to the working region. EndoVac can securely supply the irrigant's instrumenting length without extruding into the periapical region [40,41]. Rinsendo is a pressurized suction device that operates at about 100 rpm [42]. It consists of a cannula and a handpiece with an exit orifice. The irrigation speed is 6.2 mL/minute and is operated by an air compressor. This system involves drawing 65 mL of a 1.6 Hz oscillating rinsing solution from a syringe and transferring it to the root canal using a specially designed cannula. The Rinsendo system underwent evaluation using a split tooth model, revealing its effectiveness lower than manual dynamic irrigation [43].

Photoactivated disinfection

Endodontic irrigation with photoactivated disinfection (PAD) aims to reduce any bacteria in the root canal. The photosensitizer (PS), when coupled with oxygen, produces cytotoxic species and is used in the PAD technique in conjunction with low-intensity visible light. The bacteria perish as a result of this breach in their cell wall. Furthermore, PAD is effective against protozoa and viruses [44,45]. The PS attaches itself to the surface of microorganisms and binds there. It is a highly diluted form of toluidine blue O. It takes up light energy, releases oxygen, and then changes into highly reactive oxygen radicles and ions [46].

Ozone-based delivery system

Three oxygen atoms make up the triatomic molecule known as ozone. It is applied topically as oxygen/ozone gas and ozonated water. As a result of its instability and simple dissociation again into oxygen (O_2_), singlet oxygen (O1), a strong oxidizing agent that additionally harms microorganisms, is released. Several delivery systems for endodontic irrigation include the OzoTop, Heal Ozone (Kavo), and Neo Ozone Water-S units. Ozonated water was shown by Nagayoshi et al. to be very effective at eliminating gram-positive and negative microorganisms [41,47].

Laser

Recent research has shown that pulsed energy from lasers can activate irrigation solutions. This discovery suggests that using erbium, chromium: yttrium-scandium-gallium-garnet, erbium-doped yttrium-aluminum-garnet (Er:YAG) laser light in a laser-activation irrigation system may be more effective for removing dentinal debris and smear layer during dental procedures. Moreover, the antibacterial properties of NaOCl are enhanced through lasers. Er:YAG is the best laser for removing dentinal debris and smear layers, according to numerous studies. The optical fiber’s tip emits laser energy that travels along the canal rather than always lateral to the walls. Although a total removal of the bacteria and biofilm was not yet achievable, a delivery system that lets lateral radiation emission attempted to improve the antimicrobial effect to get around this restriction [48,49]. As a result, strong evidence is still lacking for the direct disinfection of root canals using high-power lasers.

## Conclusions

To properly clean and disinfect canals during a dental instrumentation procedure, it is recommended to flush a significant volume of NaOCl solution into the canals. After shaping, the canals should be thoroughly washed with citric acid or aqueous EDTA. To rinse each canal, 5-10 mL of the chelating irrigant should be used and left to run for at least one minute. Finally, giving the area a last rinse with an antibacterial solution is advised after removing the smear layer.
